# Global health is political; can it also be compassionate?

**DOI:** 10.7189/jogh.09.020306

**Published:** 2019-12

**Authors:** Beniamino Cislaghi, Paul Bukuluki, Mushtaque Chowdhury, Angelica Espinosa Miranda, Leah Kenny, Anjalee Kohli, Santi Kusumaningrum, Balkissa Harouna Brah, Catherine Love, Mahesh Madhav Mathpati, Paul Nkwi, Fernando Ona, John Porter, Mónica Ruiz-Casares, Neela Saldanha, Munshi Sulaiman, Mike Wessells

**Affiliations:** 1 London School of Hygiene and Tropical Medicine, London, United Kingdom.; 2 Makerere University, Kampala, Uganda; 3 BRAC, Dhaka, Bangladesh; 4 Universidade Federal do Espírito Santo, Vitória, Brazil; 5 Institute for Reproductive Health, Georgetown University, Washington D.C., United States; 6 PUSKAPA, FISIP University, Depok, Indonesia; 7 Geneva Global, Niamey, Niger; 8 Independent Consultant, New Zealand; 9 Catholic University of Cameroon – Bamenda, Bamenda, Cameroon; 10 Tufts University, Boston, United States; 11 McGill University, Montreal, Canada; 12 Ashoka University, Sonipat, India; 13 BRAC University, Dhaka, Bangladesh; 14 Columbia University, New York City, New York, USA

One would think that global health, as “trans-national research and action for promoting health for all” [1], should be rooted in the compassionate desire to alleviate suffering. Yet, its current operationalisation has been criticised for bureaucratising action into addressing technical (rather than moral) problems. This bureaucratisation doesn’t help global health researchers and practitioners resist a natural sense of disconnect with people living in very distant places [2]. As Addis [2] noted, compassionate responses to global health issues both require recognising suffering as such and demand purposeful action. Ethical questions for compassionate global health practitioners then become: how do we identify (as opposed to assume) people’s suffering, and what should we do to alleviate it, without making it worse?

Interventions for health promotion and harm prevention are ethical mine fields. While several commentators have offered important ethical insight into the different approaches to health promotion [3], less reflection has gone into whether public health institutions have a mandate to promote health in the first place. Those who did look at the debate on responsibility for public health often mention Mill’s harm principle: one’s freedom can be limited only to prevent harm to others [4]. A public health intervention would thus be justified to, for instance, try to stop people from smoking – even if that happened in private without hurting anyone else –because a national health system couldn’t afford having a large majority of the tax-paying middle-age population in hospital for the rest of their life. Yet, this feels wrong. Don’t we have a responsibility for mutual care, including protecting people from self-harm? The general consensus is that yes, we do, as demonstrated, for instance, by the global public health efforts conducted to prevent suicide [5].

An alternative solution to the conundrum at the health/freedom nexus lies in the suggestion that the harm principle doesn’t apply to public health interventions because these interventions don’t affect people’s freedoms. Several of these interventions formally commit to increase people’s capacity to make informed choices: whether people take up a new healthier behaviour or not – once they are aware of the consequences – is up to them. This answer, however, is not quite satisfactory either, as it lies on an illusionary belief in people’s freedom to choose as if they were not conditioned by the social, political and economic context. Most public health interventions are embedded within systems of values that affect what information and options are presented to people (for instance, suggesting condoms as a solution to the spread of HIV rather than abstinence, or vice versa), in ways that profoundly inform and even shape their decisions. Is there a valid justification, then, for public health interventions that aim to keep individuals and communities from doing what is harmful to them?

Global health programmes are not safe from these criticisms; if anything, things get muddier there. Granted, the cross-cultural prevention of harmful traditional practices (such as female genital mutilation/cutting, child marriage, breast ironing or forced feeding) seems to be simpler to tackle, at least from a rights-based approach: If the victims of those practices don’t want to comply with them, why should they? It shouldn’t thus matter whether the practice has benefits for the group (eg, it strengthens existing social relations or ensures the continuation of family lineage): if the individuals who are supposed to undergo it don’t want to, that alone justifies an intervention. Many interventions, then, try to increase victims’ resilience and resistance against perpetrating powerholders that are enforcing these practices on them. Here, the underlining working assumption is that the people who undergo a harmful practice (the victims), if given the choice, would choose not to. Since they are victims because they are not really free, helping them become free will ensure they are not victims anymore.

Whether this assumption is always correct, is not easy to say. In other public health fields, solid evidence exists that people do not always make the “healthy” choice, when they are, supposedly, free to do so. In their multi-country study, Banerjee and Duflo [6], for instance, notoriously showed that poor people who received food vouchers did not buy more of the usual affordable food (eg, rice) they consumed. Rather, they purchased junk food (eg, chocolate bars), obviously failing to achieve the nutritional outcomes that the intervention aimed for. In cross-cultural work on harmful and gender-related practices, many interventions are still being designed with an assumption that expanding people’s agency – their capacity to “define life goals and act upon them” [7] – will reduce their compliance with a harmful practice. This might seem logical. After all, while in the rice example people might have needed to understand the basics of how food intake affects health, in the case of a harmful practice the desire to stop suffering should not require any scientific knowledge: presented with the opportunity, people will just do the ‘right’ thing. Why would someone freely choose to do something that is harmful to them or that restricts their freedoms?

## A CASE STUDY: ADOLESCENT-LED MARRIAGE IN SOMALIA

An answer to this question comes from the findings that surprised some of the authors of this opinion piece, as they were conducting a qualitative study on child marriage in four districts in Somalia [8]. In their data, they noticed an interesting line of inquiry: adolescents’ increased access to smart phones expanded opportunities for private unsupervised conversations with people of the opposite sex. In a context where premarital sex was unacceptable and where marriage was considered ‘cool’, these adolescents arranged secret meetings on the internet that eventually culminated in them having sex and eventually eloping. These Somali participants reported that parents no longer had control over whom their children talked to. It used to be parents who had the final say on when and to whom their children would marry, often with protective effects. Now, instead, adolescents could secretly leave the household and, after a couple of nights spent with their lover, come back home and tell their parents that they had slept together. Would the parents accept and approve of their union, allowing them to get married, or would they face community humiliation that their child disobeyed them and had premarital sex (which would possibly also reduce their child’s future marriage prospects)? Most parents in these communities obviously consented to these unions (although they often disapproved them), allowing their adolescent children to get married.

## AGENCY, FREEDOM, SOCIAL NORMS, AND GLOBAL HEALTH

The Somali adolescent girls who lived in these communities used their expanding agency to get married against their parents’ will. Even though we do not intend to suggest that the global dynamics of child marriage have shifted to the point that most of it is now adolescent-led, the Somalia example is not an isolated case either. In rural Honduras, Murphy-Graham and Leal found evidence that technology had changed dating rules, increasing girls’ capacity to exercise their agency and eventually resulting in their marriage [9]. In Nepal, Human Rights Watch found a surprising percentage of marriages initiated by children [10]. In Guatemala and Brazil, Taylor and colleagues found a similar dynamic, with several children deciding spontaneously to get married [11]. And in South East Cameroon, Shakya and colleagues also found that expanded access to internet was increasing the prevalence of child marriage in the region [12]. To put it bluntly: adolescents that could now choose for themselves when to get married were deciding to do it at an age when international organisations judge it harmful for them to do so. Granted, many of them did so to leave an abusive household, to escape poverty, to have some control over their marriage before their parents decided for them, or to follow existing social norms that assigned higher status to married adolescents. From their perspective, getting married was indeed the right thing to do. The logical implication for interventions, one might conclude, is that expanding agency is not enough to achieve change – one has also to tackle other institutional, material and social factors that sustain a harmful practice. Yet, one is left wondering, to what extent can changing the social and material features of a context be justified as an attempt to increase people’s freedoms and opportunities, and when does it instead become social engineering – enforcing new practices onto people through endless nudges, rewards and incentives. We struggle to find in Mill’s harm principle a value-neutral justification for similar cross-cultural interventions.

**Figure:**
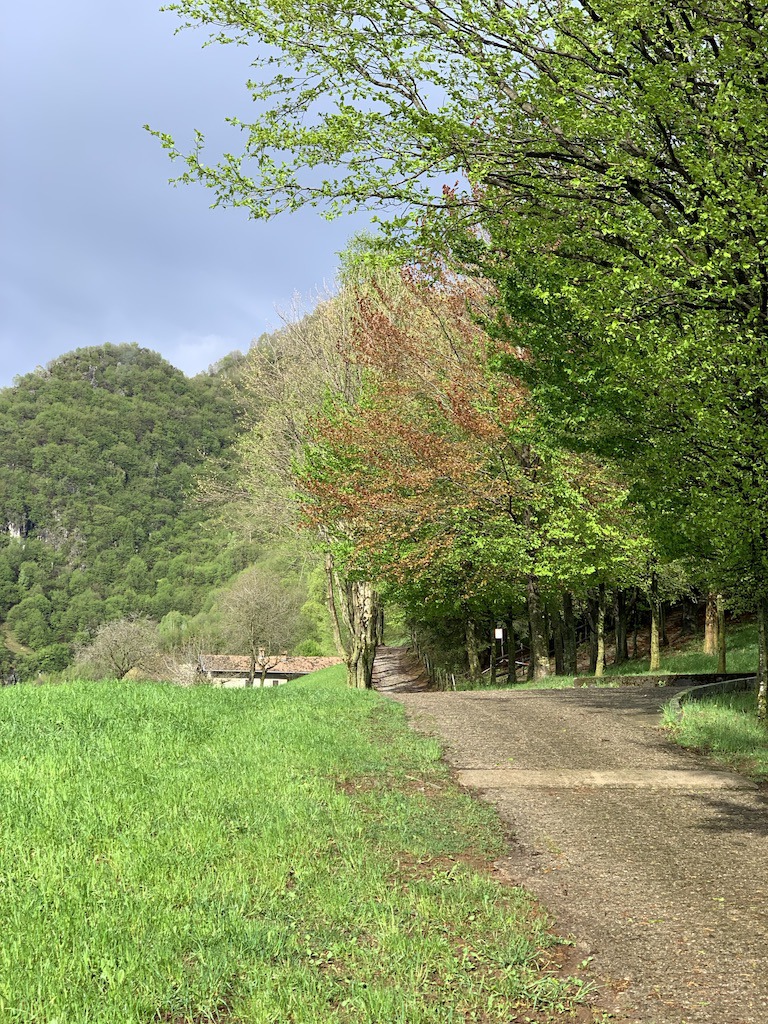
Photo: The road towards a value-based approach to global health (from the collection of Ben Cislaghi, used with permission).

## BRINGING COMPASSION INTO GLOBAL HEALTH

Ultimately, when it aims to reconfigure the social and material conditions in which people live, global health is a social and a political endeavour neither value-neutral or technical. This political aspect of global health demands to consider its potential imperialistic nature: if global health interventions are accepted as value-based (rather than simply justified by the seemingly value-neutral language of health), a question then arises as to whether they are overriding the values of people they reach out to, which would obviously make it inconsistent with compassionate action. We argue that global health practitioners, aware of the values informing their work as well as the power imbalances embedded in cross-cultural interventions from the Global North to the Global South, can draw on processes and methods that invite partners to join into inclusive, power-aware and value-based conversations. Those conversation could potentially allow them to discover that they share some of those fundamental, corner-stone values (even some that might sustain harmful practices). This will lead, overtime, to build and rebuild shared normative systems that embody visions and goals of cross-cultural health interventions, at the global and local levels. Practitioners implementing compassionate global health interventions accept the political nature of their work and are aware of the values that guide them. At the same time, they open dialogue on those values, accepting the possibility that these conversations might challenge and transform how both parties see global health, revealing new pathways of action to walk together. Compassionate global health actors thus design value-informed interventions with the hope of alleviating suffering, but remain open to the possibility of being profoundly challenged on both what people feel and the actions required to alleviate their suffering. There is much to be learnt from cross-cultural conversations and disagreements on the fundamental (and yet shifting) values informing people’s health-related practices. Inclusive, informed discussion about what is in people’s best interests (and particularly in the interest of vulnerable people) can help us achieve greater well-being for all.

We need a renewed vision for global health as well as a renewed vision of ourselves in it. If, as researchers and practitioners, we spend too much time protecting our careers and our funding sources at any costs, and too little time with the people we aim to help, we will struggle to both recognise their suffering as suffering and accept the extent to which we can be profoundly mistaken in what these people need. To improve health for all, we need to create global health systems that help us, or even demands of us, to feel the true suffering of those women, men, girls, and boys that these systems aim to help. We look forward to a value-based approach to global health that aims to do things *with* people, rather than *to* people.
